# Feasibility study on stereotactic radiotherapy for total pulmonary vein isolation in a canine model

**DOI:** 10.1038/s41598-021-91660-y

**Published:** 2021-06-11

**Authors:** Ji Hyun Chang, Myung-Jin Cha, Jeong-Wook Seo, Hak Jae Kim, So-Yeon Park, Byoung Hyuck Kim, Euijae Lee, Moo-kang Kim, Hye-sun Yoon, Seil Oh

**Affiliations:** 1 Department of Radiation Oncology, Seoul National University Hospital, Seoul, Korea; 2 Division of Cardiology, Heart Institute, Asan Medical Center, University of Ulsan College of Medicine, Seoul, Korea; 3 Division of Cardiology, Department of Internal Medicine, Seoul National University Hospital, Seoul, Korea; 4 Department of Pathology, Seoul National University Hospital, Seoul, Republic of Korea; 5 Department of Radiation Oncology, Seoul National University College of Medicine, Seoul, Korea; 6 Cancer Research Institute, Seoul National University College of Medicine, Seoul, Korea; 7 Department of Radiation Oncology, Veterans Health Service Medical Center, Seoul, Korea; 8 Institute of Radiation Medicine, Seoul National University Medical Research Center, Seoul, Korea; 9 Department of Radiation Oncology, Seoul Metropolitan Government-Seoul National University Boramae Medical Center, Seoul, Korea; 10 Division of Cardiology, Department of Internal Medicine, Sejong General Hospital, Bucheon, Korea; 11 Department of Internal Medicine, Seoul National University College of Medicine, Seoul, Korea

**Keywords:** Cardiology, Preclinical research, Translational research, Medical research, Experimental models of disease, Arrhythmias, Atrial fibrillation

## Abstract

We tested the feasibility of pulmonary vein (PV) and left atrial (LA) posterior wall isolation using non-invasive stereotactic ablative body radiotherapy (SABR) and investigated pathological changes in irradiated lesions in a canine model. Seven male Mongrel dogs received single-fraction 33 Gy SABR. We designed the en-bloc circular target of total PVs and LA posterior wall to avoid the esophagus. The circular box lesion included the LA roof and ridge, low posterior wall, and posterior interatrial septum. At 6 weeks or 4 months post-SABR, electrical isolation of the SABR lesion was confirmed using LA posterior wall pacing, and histopathological review was performed. Electrical isolation of all PVs and the LA posterior wall was achieved in three of five dogs in the 4-month group. There was one target failure and one sudden death at 15 weeks. Although two dogs in the 6-week group failed to achieve electrical lesion isolation, the irradiated atrial myocardium showed diffuse hemorrhage with inflammatory cell infiltration. In successfully isolated 4-month model dogs, we observed transmural fibrotic scarring with extensive fibrosis on irradiated atrial tissue. The findings suggest that this novel circular box-design radiotherapy technique using SABR could be applied to humans after further studies are conducted to confirm safety.

## Introduction

Atrial fibrillation (AF) is the most common cardiac arrhythmia, which increases the risk of stroke, hospitalization for heart failure, and death^1^. Catheter ablation is a well-established interventional approach for treating drug-refractory AF, but it is technically demanding, time-consuming, and invasive. Nevertheless, recurrence after catheter ablation occurs in at least 20–40% of patients, and there are complication risks related to the procedure in patients with advanced age or co-morbidities^2,3^. To overcome these limitations and invasiveness of the procedure, there is a need for a simple but effective energy delivery method. Recently, stereotactic radioablation has been suggested as a possible optional treatment for cardiac arrhythmias^4^.

The myocardial sleeve of the pulmonary vein (PV), which is known to be responsible for AF initiation, is the target of the ablation procedure^5^. Moreover, left atrial posterior wall isolation is known to reduce the recurrence of AF^6^. Therefore, the main treatment for AF ablation is electrical PV isolation (PVI) and posterior wall isolation (if needed) using an energy-delivery catheter technique. The objective of the AF ablation procedure is to create transmural, continuous, and permanent cellular damage in the targeted area.

Recently, stereotactic radioablation, which is known as stereotactic body radiotherapy or stereotactic ablative body radiotherapy (SABR) in the radiation oncology field, has shown great efficacy with acceptable toxicity in the treatment of refractory ventricular tachycardia (VT)^7^. However, SABR for AF treatment has only been attempted on a very limited number of patients due to the close proximity of the PVs to other organs, especially the esophagus^8, 9^. Previous in vivo animal studies on PVI with irradiation have shown the possibility of applying radiotherapy to PVI^4,10–14^, but these studies targeted only a single PV, which was the right superior PV ostium in most cases because of its sufficient size and study feasibility.

In the current study, we aimed to assess the feasibility of PVI with posterior wall isolation using a SABR technique in a canine model.

## Methods

Our experiments were approved by the Institutional Animal Care and Use Committee of Seoul National University Hospital (approved number: 18-0114-S1A1) and the animals were maintained in an Assessment and Accreditation of Laboratory Animal Care (AAALAC) international-accredited facility (#001169) in accordance with the Guide for the Care and Use of Laboratory Animals 8th edition. This study was carried out in compliance with the ARRIVE guidelines^15^.

### Animals

Seven adult male Mongrel canines weighing 25–30 kg with baseline sinus rhythm (SR) were included in the study. A dog model was chosen since canine cardiac anatomy and electrophysiology are similar to that in humans and have been widely used in arrhythmia researches^16^. Among seven dogs, three were randomly selected for the persistent AF model. The other four dogs were assigned to the SR model. Afterwards, one dog from the AF model and one dog from the SR model were randomly selected to harvest at 6 weeks after irradiation and the remaining five dogs (two AF model dogs and three SR model dogs) were harvested at 4 months.

### Sinus rhythm and AF models

Dogs with SR were monitored with an implantable loop recorder (Reveal LINQ, Medtronic Inc., Minneapolis, MN, USA) to monitor cardiac rhythm. The AF burden before treatment was less than 0.1% of the total monitoring duration. Two dogs (4-month cases) could not be continuously monitored because of a technical problem (battery status).

Persistent AF was induced in three randomly chosen dogs using rapid atrial pacing of the left atrial appendage for 1 month. The dogs were anesthetized and the chest was opened via minimal left thoracotomy at the fourth intercostal space. A bipolar screw-in pacing lead was fixed on the left atrial appendage via a minimal pericardial incision. The lead was then connected to a cardiac implantable electronic device (Unify Quadra or Promote RF; St. Jude Medical, Sylmar, CA, USA) placed in a pocket between the left chest muscle layers. The chest was closed, and the dog was allowed to recover for approximately 1 month before pacing was continued. Rapid atrial pacing was performed with 600 b.p.m. (pulse width 1.0 ms; 3.0 V) at 10 Hz. The dogs were followed on a weekly basis. The pacing was turned off and cardiac rhythm was checked to determine if the dog had developed AF. After 1 month of pacing, all three dogs developed AF that was sustained for more than 48 h. The dogs were observed for an additional 2 weeks before radiotherapy, and two of them were found to have sustained AF, but one dog was switched to the SR model on the 7th day from pacing-off, and further rapid pacing for 7 days was performed.

### Computed tomography (CT) simulation

Under general anesthesia, dogs were placed on a vacuum cushion in a prone position. In the first two dogs, immobilization using a thermoplastic mask was used; however, due to immobilization failure with the thermoplastic mask, the immobilization device was changed to a vacuum cushion. Contrast-enhanced CT simulation was performed after marking the reference point (1.8 ml/s, 80 ml, 10–12 s). All dogs were scanned using the Brilliance CT Big Bore (Phillips, Cleveland, OH, USA). In dogs with a large respiratory amplitude (more than 5 mm), respiratory gated 10-phase four-dimensional (4D) CT images were acquired (RPM; Varian Medical Systems, Palo Alto, CA, USA). In dogs with a minimal respiratory amplitude less than 5 mm, a single-phase scan was performed. The slice thickness was 1 mm.

### Target contouring and treatment planning

**Figure 1:**
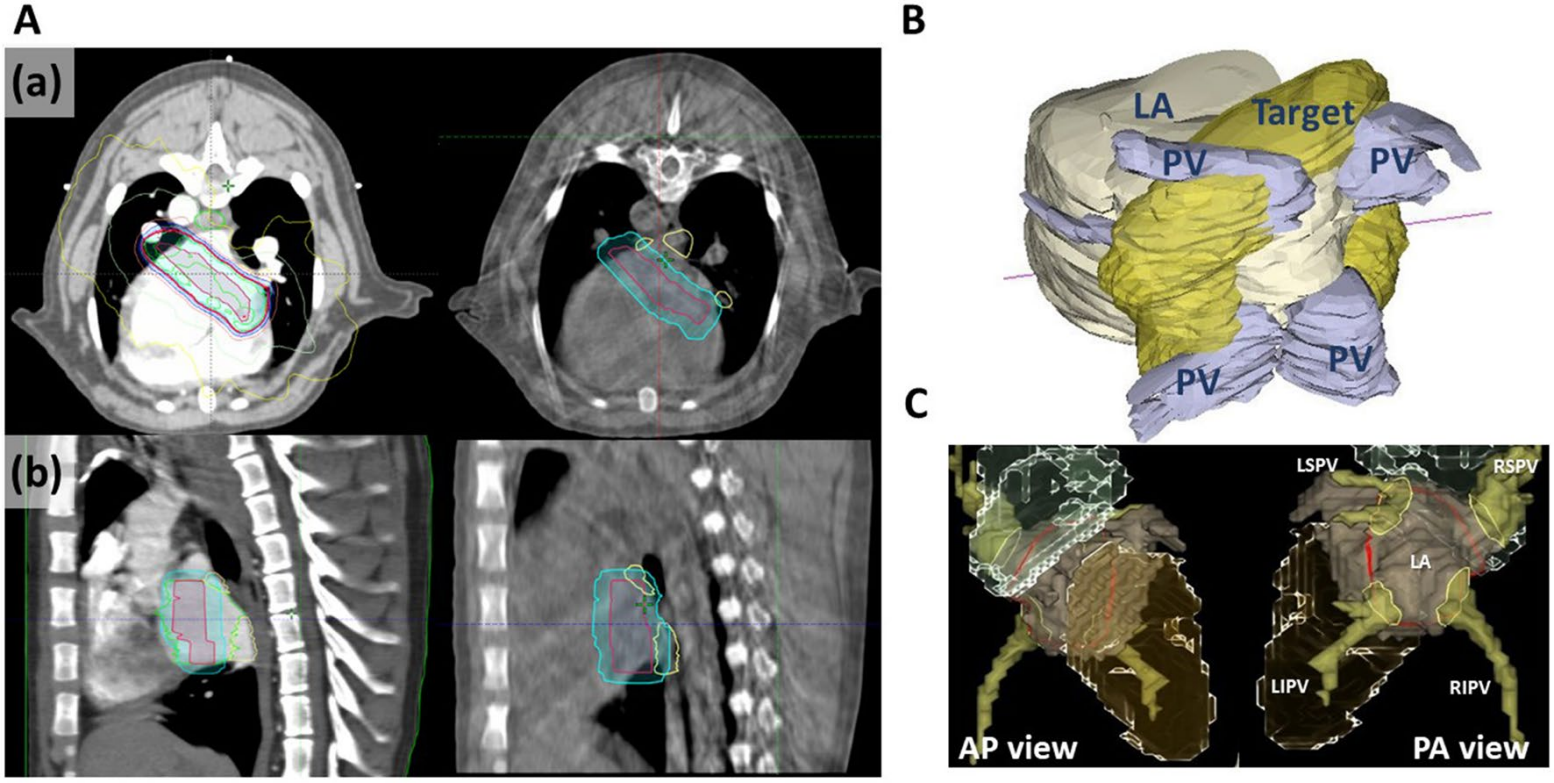
Radiotherapy design. (**A**) Axial (a) and sagittal (b) views of simulation CT with dose distribution with isodose lines (left), and cone beam CT for set-up verification (right). Red, target; cyan, planned target volume (PTV); yellow, pulmonary veins; light green, left atrium. (**B**) Posterolateral view of 3D reconstructed contoured target. (**C**) Three- dimensional reconstruction images from PA and AP views using CARTO-3 mapping system. The red line indicates the border of circular box lesion. *CT* computed tomography, *LA* left atrium, *PV* pulmonary vein, *LSPV* left superior pulmonary vein, *RSPV* right superior pulmonary vein, *LIPV* left inferior pulmonary vein, *RIPV* right inferior pulmonary vein.

The SABR target for PVI was a “box lesion” in which all PVs as well as the posterior left atrial wall were encompassed by one large thick encircling belt lesion, which is used in all Maze procedures^17^. Largely, the target was delineated in two ways. First, in the initial two cases, the internal target volume (ITV) was delineated in a circle border-like donut shape at the left atrium (LA) wall that included four PV ostia inside the circle based on 4D-CT, and a 1-mm planning target volume (PTV) margin was added to the ITV). In these initial two cases (two dogs with SR followed for either 6 weeks or 4 months), we experienced difficulties in cone beam CT (CBCT) matching because along with immobilization issues, uncertainty had increased because the ITV was confined to the LA wall and small PTV margin. Therefore, our subsequent target was made in a full circle-like shape, and a 4–5-mm margin was added to the ITV (Figs. 1A and 1B). Additionally, for CBCT matching, normal organs including the esophagus, trachea, bronchus, and pulmonary vessels were delineated. A schematic target line on a 3-dimensional reconstructed cardiac image is presented in Fig. 1C.

Treatment planning was performed with Eclipse software (Varian Medical Systems, Palo Alto, CA, USA). The prescribed dose was 33 Gy in a single fraction. The main goal of the plan was to provide 100% volume of the PTV with administration of at least 99% of the prescribed dose. The purpose of the present work was to assess the feasibility of pulmonary vein isolation using a box design, and to enhance feasibility, safety measurements were a secondary consideration in the current experiment. Detailed dosimetric data on the planning results are presented in Supplementary Table S1.

### Radiation treatment

SABR was delivered using TrueBeam (Varian Medical Systems, Palo Alto, CA, USA) under anesthesia. Thorough CBCT matching was performed for an alignment check before SABR. The radiation energy was delivered under the dog’s intrinsic rhythm without cardioversion (i.e. under a SR in the SR group and under AF in the AF group).

### Post-treatment electrophysiologic evaluation

After the planned time period (6 weeks or 4 months), all dogs were premedicated with tiletamine–zolazepam (Zoletil; Virbac, Auckland, New Zealand) 5 mg/kg. After endotracheal intubation, general anesthesia was administered using isofluorane/O_2_, and respiration was maintained with a mechanical ventilator. The chest was opened via a median sternotomy and the pericardium incised. The right atrial appendage, left atrial appendage, and left atrial posterior wall were paced using two distal electrodes on a 5-Fr quadripolar electrophysiology catheter (Supreme, St. Jude Medical, Minnesota, MN, USA). To capture the atrium, high-output pacing with a pacing cycle length of 1000 ms was performed from 10 to 0.1 mA. A “box lesion” conduction block was defined when the atrium was not captured under supraphysiologic amplitude (five times the threshold of the left atrial appendage)^18^. The pacing test was conducted independently by two researchers (M.J.C and M.K.K).

One dog (SR/4-month) underwent pre- and post-radiotherapy electroanatomical 3-dimensional mapping of the LA using CARTO XP (Biosense Webster Inc., Diamond Bar, CA, USA). Using roving of the catheter tip, local electrical amplitudes were determined in the right upper PV antrum, proximal vein, and distal vein, and an electroanatomic voltage map was created. Voltage amplitudes of 1.0 mV indicated viable tissue. Voltages of 0.5 mV were considered representative of scar tissue. The proximal vein represented the target area for radiation.

### Anatomical pathologic evaluation and immunohistochemical analysis

Gross and microscopic pathology reviews were performed by a cardiac pathologist (J.W.S). After electrophysiological evaluation, the heart, including the pericardium, was explanted for gross examination. The heart and esophagus were stored in 10% formalin. Coronal or sagittal sectioned paraffin-embedded block tissue (size: 1 × 2 cm) was obtained from the targeted area with nearby tissue. Hematoxylin and eosin (H&E) and Masson's trichrome (MT) staining were performed for histopathological evaluation. For the immunohistochemical analysis of Connexin-43 (Cx-43), paraffin blocks were re-cut and stained with the Cx-43 monoclonal antibody (Invitrogen#13-8300). Digital microscopic images were analyzed using Aperio Imagescope (Leica Biosystems, Buffalo Grove, IL, USA).

## Results

### Follow-up

Of the total seven dogs, six dogs survived until harvest day, but one dog died 1 week before its intended harvest day (SR, 4-month follow-up model). An immediate autopsy was performed, and we found there was a scant amount of clear yellowish pericardial effusion without the pericardial tamponade feature. No evidence of coronary artery obstruction, perforation, intracardiac thrombus, or pulmonary embolism was observed. The diameter of the four cardiac chambers was within normal limits. The irradiated lesion was fibrotic, but the other part of atrium near the sinus or atrioventricular nodes was preserved. Cardiac rhythm was not monitored at that timepoint, and therefore, we could not determine the cause of sudden death.

Finally, we analyzed six dogs (two dogs with 6-week follow-up and four dogs with 4-month follow-up) for irradiated lesion isolation with pacing test. All six dogs maintained their initial rhythm until harvest. However, the AF burden of one dog in the AF group decreased from > 99 to 83% after radiotherapy, although the AF burden of one dog with SR increased from < 1 to 3% after radiotherapy. The dogs with AF on the harvest day were switched to the SR model with direct current cardioversion before the pacing test. During the follow-up, fatal tachy- or brady-arrhythmia were not observed upon continuous ambulatory monitoring.

### Pulmonary vein and left atrial posterior wall isolation

**Figure 2:**
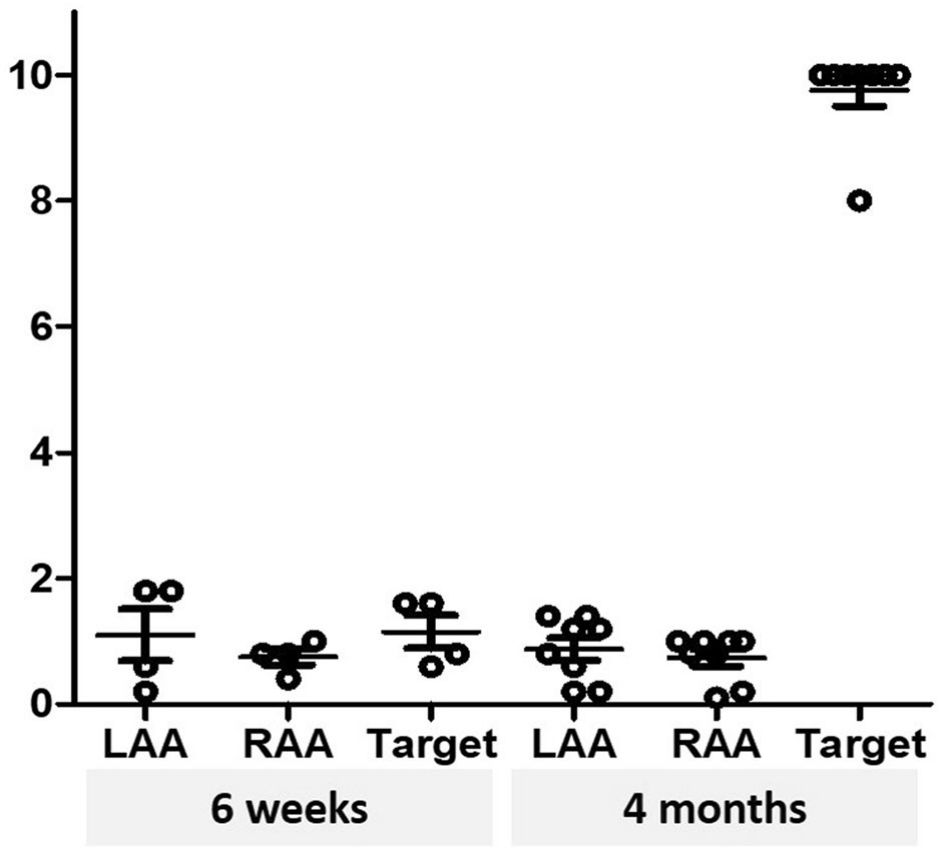
Post-radiotherapy cardiac rhythm and electrophysiologic evaluation. Atrial pacing tests confirmed that pacing was not captured inside the irradiated target area at 4 months. *LAA* left atrial appendage, *RAA *right atrial appendage.

**Figure 3:**
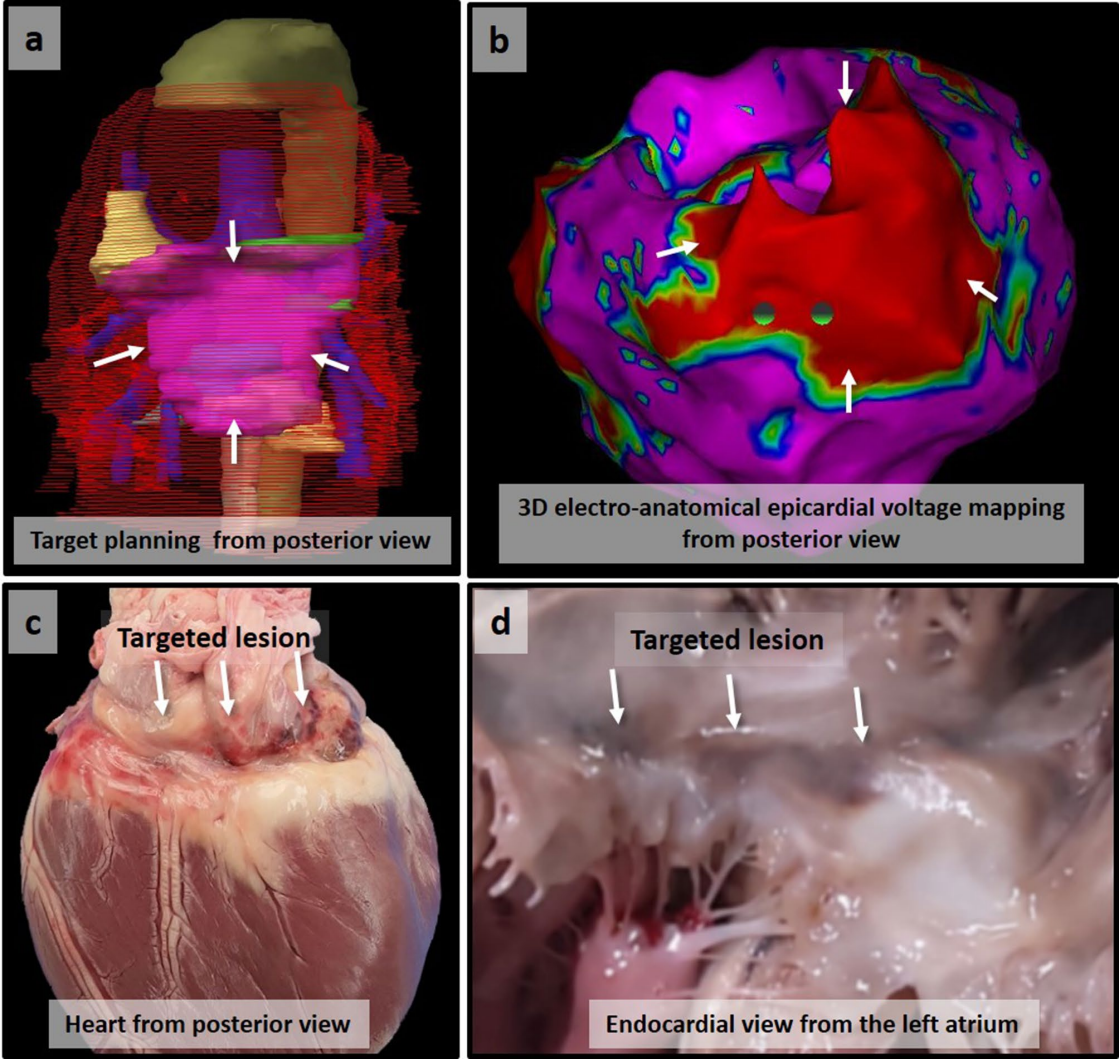
Myocardial necrotic scar formation on irradiated left atrial posterior wall. The reconstructed target area from the posterior view (white arrows in **a**) corresponds with the low voltage area (red area with white arrows in **b**) indicating a scarred lesion on voltage mapping with the CARTO-3 electroanatomical mapping system (Biosense Webster Inc.). The targeted lesion is grossly distinguishable at 4 months from both the epicardial (**c**) and endocardial (**d**) side.

As demonstrated in Table 1, a conduction block from the left atrial posterior wall to the atrium was achieved in 0% (0/2) in the 6-week group and in 75% (3/4) in the 4-month group (except for the one dog that died suddenly). In three successful cases, pacing from the posterior wall inside the target lesion did not propagate outside the target area. The repeated pacing threshold test results showed that capture failure was confirmed inside the irradiated target area at 4 months (Fig. 2). In the failed cases, the pacing threshold was higher in the AF model than the SR model. The one failed case in the 4-month group had undergone different radiotherapy planning, as described in the Methods section. The low voltage area indicating a scarred lesion on voltage mapping with the CARTO-3 electroanatomical mapping system (Biosense Webster Inc.) was consistent with the radiotherapy target area corresponding to the planned target for irradiation (Fig. 3a,b).

**Table 1:** Study results.

Follow-up	Initial	Cardiac rhythm		Pacing threshold (mA)^a^		Study result	
		Harvest day	Inside IR lesion (LA posterior wall) (**A**)	Left atrial appendage (**B**)	Right atrial appendage	**A/B**	
6 weeks	SR	SR	0.8	0.8	0.8	1	Fail
6 weeks	AF	AF^b^	1.6	1.8	0.8	0.89	Fail
4 months	SR	SR	> 10	0.2	0.2	> 50	Fail
4 months	SR	–	–	–	–		N/A
4 months	SR	SR	> 10	0.8	0.8	> 12.5	Success
4 months	AF	AF^b^	10	1.4	1.0	7.14	Success
4 months	AF	AF^b^	8	0.8	1.0	10	Success

*SR* sinus rhythm, *AF* atrial fibrillation, *IR* irradiated, *LA* left atrial, *N/A* not applicable.

^a^Pulse width 1.0 ms.

^b^Pacing threshold test was performed under sinus rhythm after direct current cardioversion.

### Gross lesion and histopathological findings

The pericardial sac membranes of all seven dogs were thin and translucent, containing a scant amount of clear and serous fluid with no evidence of pericardial disease. The pericardial organs were visually intact without abnormal findings. There was no intracardiac thrombus in any of the seven dogs. No PV stenosis was observed in the gross examination and peri-vascular fibrosis around the coronary sinus and the coronary arteries was present without significant stenosis. The target adjacent structures such as the cardiac valves, esophagus, and aorta were intact in both the 6-week and 4-month groups (Supplementary Fig. S1 online).

There was no gross visual lesion on the target area at 6 weeks, but we definitely observed a transmural fibrotic lesion in the area corresponding to the target area at 4 months (Supplementary Fig. S2 online), which also corresponded to the planned target lesion and low voltage area on electroanatomical mapping (Fig. 3c,d).

**Figure 4:**
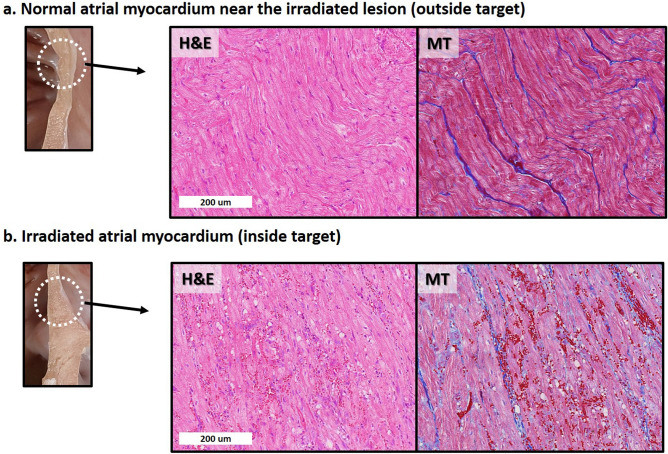
Histologic changes after irradiation at 6 weeks. There is no gross lesion at 6 weeks. However, from the light microscopic evaluation with H&E and MT staining, compared to normal atrial myocardium near the irradiated lesion (**a**), irradiated atrial myocardium (**b**) shows diffuse vacuolization with extensive hemorrhage and inflammatory cell infiltration with dilated capillaries. *H&E* hematoxylin and eosin, *MT* Masson’s trichrome.

**Figure 5:**
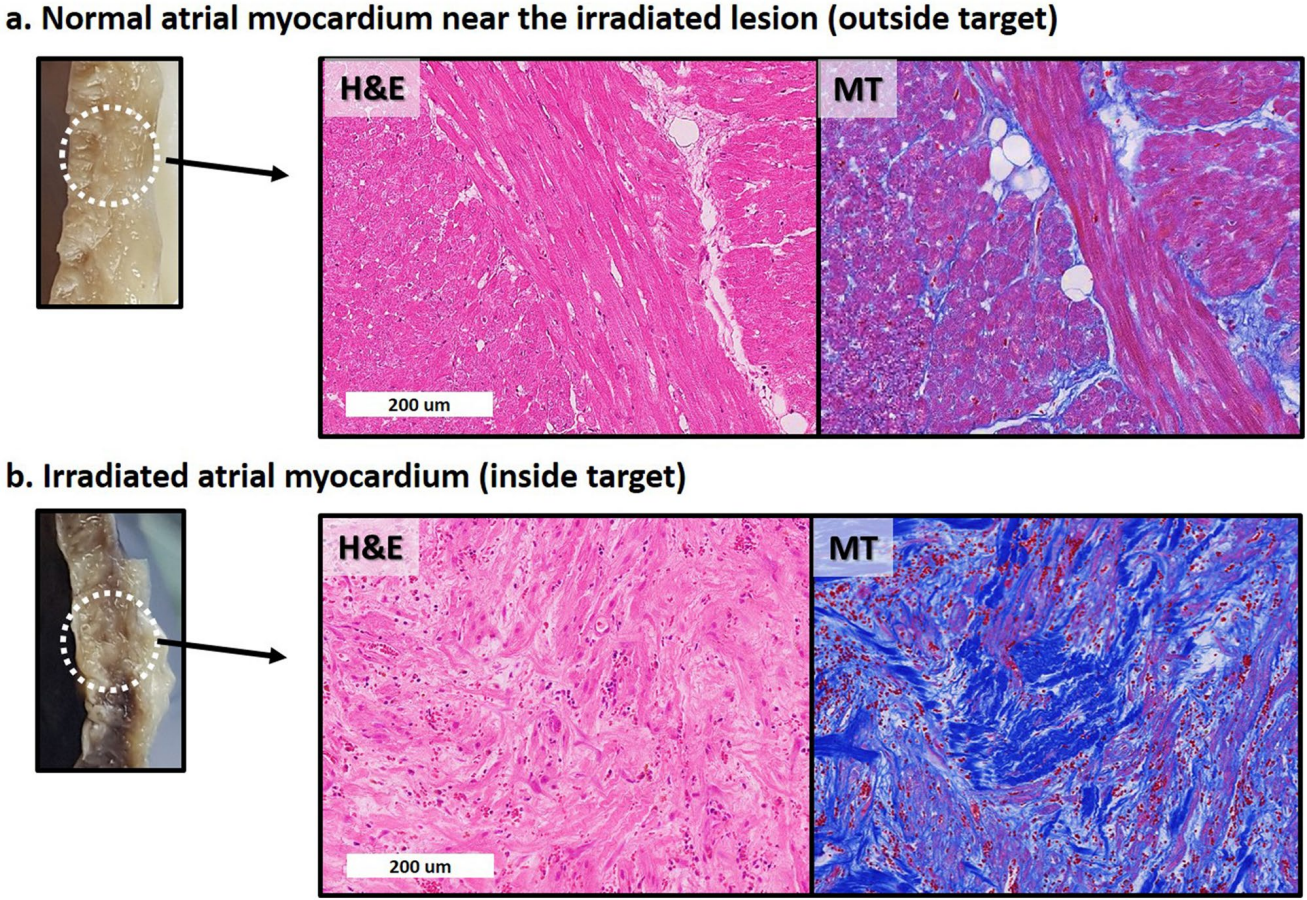
Histologic changes after irradiation observed at 4 months. A visually definite transmural necrotic lesion is observed on the irradiated area at 4 months. Compared to the normal atrial myocardium near the irradiated lesion (**a**), irradiated atrial myocardium (**b**) shows massive hemorrhage with anucleated wavy fibers with extensive interstitial fibrosis. *H&E* hematoxylin and eosin, *MT* Masson’s trichrome.

On the light microscopic evaluation at 6 weeks, compared to the adjacent myocardium outside the radiotherapy target lesion, sporadic vacuolization, diffuse hemorrhage, and inflammatory cell infiltration with dilated capillaries were observed (Fig. 4). On the light microscopic evaluation at 4 months, the target lesion was composed of massive hemorrhage with anucleated wavy fibers and extensive interstitial fibrosis (Fig. 5). The irradiated area that underwent fibrotic change showed decreased Cx-43 expression (Supplementary Fig. S3A online). The decreased Cx-43 expression in the border zone between the target area and the unirradiated myocardium was not definite at 6 weeks, but was decreased at 4 months (Supplementary Fig. S3B online). The border zone also showed a mixture of viable myofibrils and a fibrotic area with extensive hemorrhage (Supplementary Fig. S4 online).

## Discussion

To our knowledge, the present study is the first study to demonstrate the feasibility of total PV with LA posterior wall isolation with high-dose radiation in a large animal model. With a novel circular target design for SABR, the conduction block from the irradiated in-target area to the outside target was completely achieved without capturing the atrial myocardium at 4 months, but not at 6 weeks.

Recently, Monroy et al. and Qian et al. reported the human experience of non-invasive stereotactic radioablation for the treatment of atrial fibrillation^9,19^. In this study, two patients with drug-refractory AF were treated with radiation with 25 Gy single fraction. Although they reported no procedure-related complications, the inevitable exposure of critical structures, such as the esophagus, and high dose to mitral valve may have been a major concern because AF is generally not directly related to sudden death. The ablation lesion set for AF treatment is complex, so this previous study was too small to reveal the feasibility or safety of cardiac radioablation for AF.

The optimal radiation dose for PV isolation is not currently established. In the previous animal study by Bode et al.^12^ using a porcine model, right superior PV ablation was achieved using single 32.5 Gy radiotherapy. In another animal study by Zei et al., the right superior PVs of all 19 subjects (dogs or pigs) were treated with radiotherapy without complications at 35 Gy and 25 Gy with a partial effect at 20 Gy. Sharma et al.^10^ reported that the voltage decreased at the left PV ostium in a study using 16 mini swine. The above human studies on two patients also applied 25 Gy based on the previous human VT trial^20^. The most commonly used radiation dose for the treatment of VT is 25 Gy in clinical settings and this dose has shown clinical efficacy, while an optimal dose should also be set in the future. The dose-setting for AF in the current study was based on the hypothesis that the antiarrhythmic mechanisms between the two treatments could be different. In VT, antiarrhythmic effects have been observed in the early phases after radiotherapy when fibrotic scars would not be present. In AF, however, it could be speculated that the fibrotic scar should be formed for PV isolation after radiotherapy. Radiation-induced myocardial fibrosis is a well-studied change that occurs after high-dose radiotherapy and is considered as possible toxicity^21^; however, we used this mechanism for the AF treatment with a precise SABR technique. To determine the prescribed dose in the current study, we referred to the dose escalation study from Blanck et al., which showed doses > 32.5 Gy could induce circumscribed scars^11^, while Zei et al. recently reported that 25 Gy would be effective enough^14^. The goal of the current study was to assess the efficacy of SABR in AF treatment, and therefore, we selected a higher RT dose of 33 Gy rather than a dose of 25 Gy used in the VT treatment with SABR and the irradiated atrial myocardium showed transmural fibrosis.

The heterogeneity of the border zone between the irradiated and non-irradiated zones could be the arrhythmogenic substrate in the context of micro-reentry or triggered activity. However, the irradiated myocardium was well-demarcated from the adjacent normal myocardium, as displayed in Supplementary Fig. S4, and the width of the border zone was only a few hundred microns. The topic of the role of irradiated tissue as an arrhythmogenic substrate should be further elucidated in a future study.

The PVs and LA posterior wall play an important role in AF initiation and maintenance^22^. Therefore, total PV isolation with or without posterior wall isolation from the LA is the standard treatment method. However, atrioesophageal fistula or phrenic nerve palsy, which are two major complications related to AF ablation, may occur during catheter ablation of the posterior parts of the PVs^23^. Like conventional catheter ablation, high dose radiation to the esophagus could be also related to the fatal adverse event as shown in the previous report^24^. Thus, we applied a modified circular box-lesion set anteriorly to all PVs and the LA posterior wall to save the esophagus and enhance target coverage. This novel target circular lesion included the posteroinferior part of the LA, part of the atrial septum, anterior LA roof, and the Coumadin ridge area between the LA appendage and the left PVs, which are traditional target areas for conventional AF ablation. We applied this design in both SR and AF models. The AF burden of one dog in the AF model was decreased after radiotherapy, although we could not verify whether this effect was a result of radiotherapy.

Although safety is a crucial issue for application of SABR in AF treatment, the purpose of the present work was to assess the feasibility of PV isolation using a box design, and to enhance feasibility, safety measurements were considered less importantly. Therefore, in the current study, we could not ensure the safety of radiotherapy for AF treatment. One dog with SR without cardiac monitoring died suddenly. This occurred 1 week before the intended harvest day. Unfortunately, we could not verify the cause of death from the autopsy results, and the association between sudden death and radiotherapy cannot be ruled out. When we investigated the heart during the autopsy, we found extensive transmural lesions across the atrium (Supplementary Fig. S5 online). The sinus or atrioventricular node area was visually and histopathologically preserved. However pump failure and a fatal ventricular arrhythmia in the context of LV acute dysfunction cannot be excluded. The overall reduction of LV function without apparent pathological structural changes was also reported by Zei et al.^14^. The study by Hohman et al.^25^ reported cases of sudden death and a clear dose response relationship with the LV dose. In the in vivo study by Bode et al., collateral damage to the AV node that resulted in an AV block after 40 Gy irradiation to RSPV was observed^26^. There were no AV blocks in the six dogs that underwent continuous monitoring in the current study. In one case, a transient AV block was seen under general anesthesia during pre-SABR monitoring; however, no additional AV block occurred in subsequent continuous monitoring (Supplementary Fig S6 online).

Minimizing the collateral cardiac damage outside the target is crucial in cardiac radioablation. A high irradiation dose on LV has been associated with depressed cardiac function. In the previous largest animal study of RSPV isolation using SABR, mild functional echocardiographic abnormalities were reported in some animals during echocardiographic evaluations at 3–6 months, despite no structural abnormalities at the histopathological examination performed immediately after^14^. Additionally, six cases of sudden death after 40 Gy proton-therapy in multiple left ventricular sites among eight pigs associated with a significant left ventricular ejection fraction depression were reported by Hohman et al.^25^. The issue of the safety of surrounding organs, including untargeted cardiac tissue, should be considered before applying this technique for human AF treatments.

There are several limitations of this study to consider. First, we did not perform the irradiation in a sham-operated canine model as a control group. The reason for this was to minimize the number of sacrificed animals due to ethical considerations. Instead, we used the adjacent atrial myocardium in the same irradiated dog as a control part, which might be different to that of any control myocardium. However, we thought that this method was sufficient for lesion comparison because we were able to observe prominent differences between the two areas in all study subjects. Second, the canine cardiac structure is known to be different from that of human hearts. Although the canine cardiac anatomy is similar to that of a human and most of the ion currents found in a human heart are present in a dog’s heart, their action potential shape and duration are known to vary. Most importantly, the anatomy of the PVs entering the LA is different in humans and dogs. For example, there are four PVs in humans, but 5–6 in dogs. In our study, the dogs also showed complex PV anatomy connected to the LA (Supplementary Fig. S7 online). Due to these anatomical variations, we experienced set-up errors as described in the Methods section. Further, unlike in humans, reproducing the same position and target matching in anesthetized animals are very challenging. The failure in one of the animals from the 4-month group could have been caused by suboptimal coverage or target matching resulting from different contouring methods and consequent treatment planning, as the contouring method was changed after the treatment of the case. All cases with the modified RT design were successful in achieving block conduction at 4 months after SABR. Considering the difficulties in the set-up process in dogs, it would be more feasible to perform PV isolation in human with the designed SABR target. Third, we did not meticulously consider safety in the current experimental setting. Because dog atria are smaller than human atria and also ventrally elongated, the unwanted part of the left ventricle and the right atrium was included in the target planning. We did not try to avoid the left ventricle and the right atrium by minimizing the target volume. Although radiotherapy can successfully ablate the atrial myocardium, we should carefully consider the safety issue before applying this technique in human AF treatment. Fourth, a lack of echocardiographic information from a follow-up after SABR is a major limitation of this study. Additionally, 4D CT was not performed in all animals, though non-4D CT cases were selected when the respiratory amplitude was less than 5 mm. Lastly, we did not analyze the dose-responsiveness of the atrial myocardium from irradiation. There are very few studies in this field, so we focused on the feasibility of radioablation for AF. Further studies are needed to confirm the correct dose for safe radioablation.

In conclusion, all PVs and the LA posterior wall can be effectively isolated with a circular box-lesion ablated by SABR (33 Gy, single fraction) in dog models. The conduction block was achieved in the 4-month model, and the irradiated lesion was definitely differentiated from the adjacent normal myocardium in the histopathological observation.

## Supplementary Information

Supplementary Information.

## Data Availability

The data underlying this article will be shared on reasonable request to the corresponding author.
