# Supplementary material for: Feasibility study on stereotactic radiotherapy for total pulmonary vein isolation in a canine model
Source: Sci Rep. 2021 Jun 11;11:12369. doi: 10.1038/s41598-021-91660-y (PMC8196028; doi:10.1038/s41598-021-91660-y)
Supplement: Supplementary file 1 — Supplementary Information. [file 41598_2021_91660_MOESM1_ESM.docx]

**SUPPLEMENTARY MATERIALS**

**to**

**Feasibility study on stereotactic radiotherapy for total pulmonary vein isolation in a canine model**

Ji Hyun Chang, M.D., Ph.D, Myung-Jin Cha, M.D. Ph.D., Jeong-Wook Seo, M.D., Ph.D, Hak Jae Kim, M.D., Ph.D, So-Yeon Park, Ph.D, Byoung Hyuck Kim, M.D., Ph.D, Euijae Lee, M.D., Moo-kang Kim, B.S., Hye-sun Yoon, B.S., Seil Oh, M.D. Ph.D.

**Supplementary Table S1.**

| Follow-up |  | 6-week | 6-week | 4-month | 4-month | 4-month | 4-month | 4-month |
| --- | --- | --- | --- | --- | --- | --- | --- | --- |
| Cardiac rhythm |  | SR | AF | SR | SR | SR | AF | AF |
| Study results |  | Fail | Fail | Fail | Sudden death | Success | Success | Success |
| **Planning target volume** |  |  |  |  |  |  |  |  |
|  | Coverage goal |  |  |  | >99% |  |  | 100% |
|  | Maximum dose (Gy) | **38.96** | **39.49** | **37.66** | **36.7** | **36.38** | **42.1** | **37.99** |
| Spinal cord (Gy) |  |  |  |  |  |  |  |  |
|  | <0.35 cm3* | 3.4 | 12.57 | 3.08 | 7.2 | 9.52 | 8.9 | 8.37 |
|  | <1.2 cm3* | 1.01 | 9.62 | 1.58 | 5.5 | 6.94 | 7.8 | 5.95 |
|  | Maximum dose | 4.92 | 13.77 | 4.01 | 8.65 | 10.9 | 14 | 8.83 |
| Esophagus (Gy) |  |  |  |  |  |  |  |  |
|  | <5 cm3* | 4.99 | 26.42 | 2.36 | 5.47 | 8.26 | 8.7 | 23.35 |
|  | Maximum dose | 15.7 | 36.95 | 13.7 | 34.02 | 27.3 | 20.8 | 36.81 |
| Left ventricle |  |  |  |  |  |  |  |  |
|  | Volume (cm3) | 100.9 | 121.7 | 146.7 | 93.3 | 107.9 | 151.5 | 105.5 |
|  | Maximum dose (Gy) | 20.8 | 38.03 | 16.1 | 36.16 | 36.08 | 38.5 | 37.05 |
|  | Mean dose (Gy) | 3.07 | 11.99 | 1.9 | 8.66 | 9.27 | 5.23 | 6.27 |
| Lung total (both) | Volume (cm3) | 2483 | 1863.3 | 1390 | 1957.8 | 1179.3 | 1884 | 2486.4 |
|  | V20 | 0 | 1.21 | 0.36 | 2 | 2.83 | 3.78 | 1.12 |
|  | V5 | 8.88 | 21.09 | 10.6 | 23.1 | 29.3 | 25.6 | 22.2 |

Supplementary Table S1 includes the dose volume parameters for the target and organ at risk in the canine model. The presented dose volume parameters in the current study are much higher than the recommended dose constraints for ventricular tachycardia treatments,^1^ however, these dose constraints might have been too greatly overestimated to directly predict toxicity in humans. These parameters might be improved with lower doses for adjacent structures in humans, as the human heart is bigger than the canine heart.

^*^Minimum doses to the most irradiated corresponding volumes of the organ

V20, the volume irradiated to 20 Gy

V5, the volume irradiated to 5 Gy

Reference

(1) Knutson NC, Samson PP, Hugo GD, Goddu SM, Reynoso FJ, Kavanaugh JA, Mutic S, Moore K, Hilliard J, Cuculich PS, Robinson CG. Radiation Therapy Workflow and Dosimetric Analysis from a Phase 1/2 Trial of Noninvasive Cardiac Radioablation for Ventricular Tachycardia. Int J Radiat Oncol Biol Phys. 2019;104(5):1114-23.

**Supplementary Figure S1.**


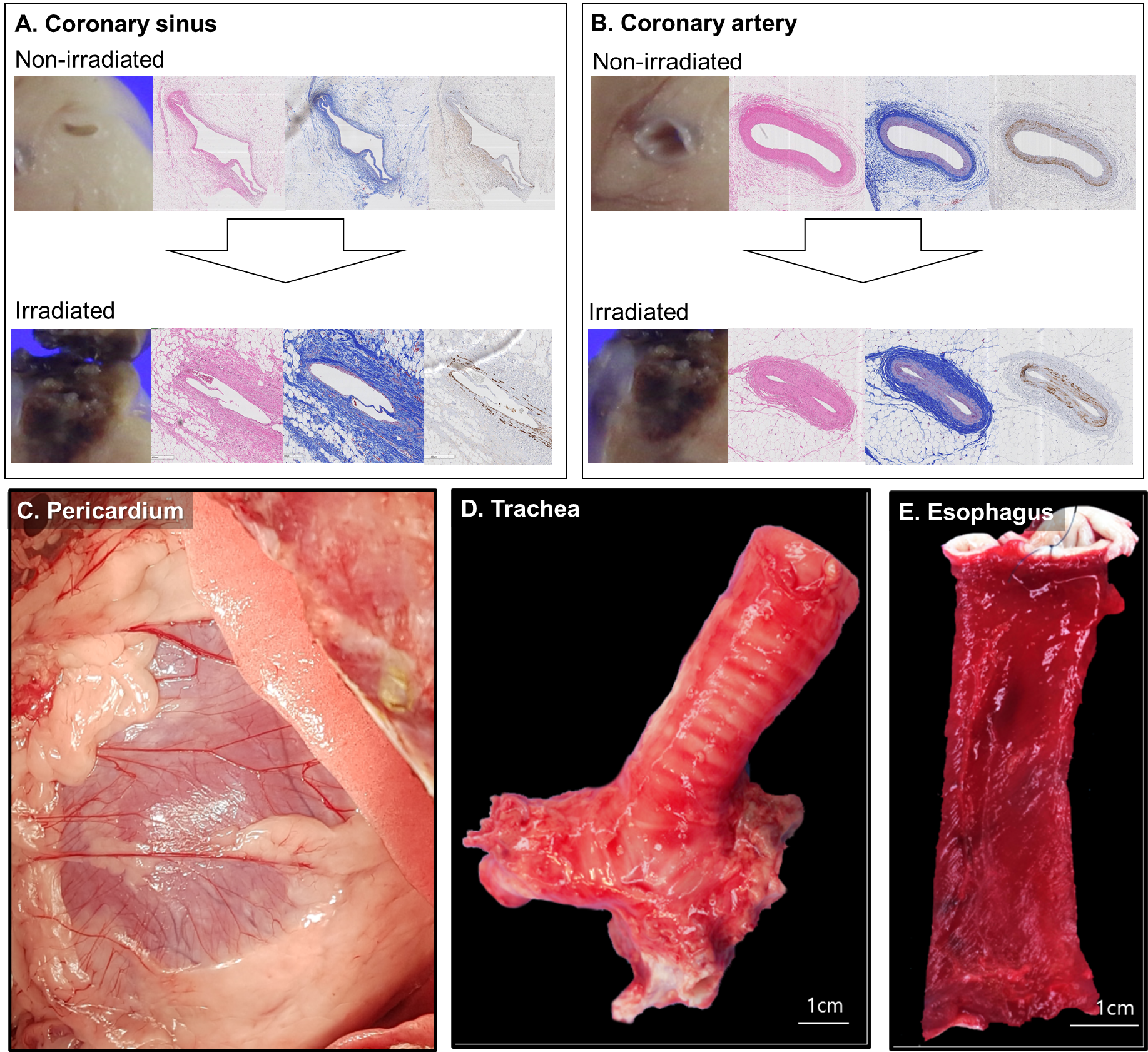


**Supplementary Figure S2.**

A. 6 weeks


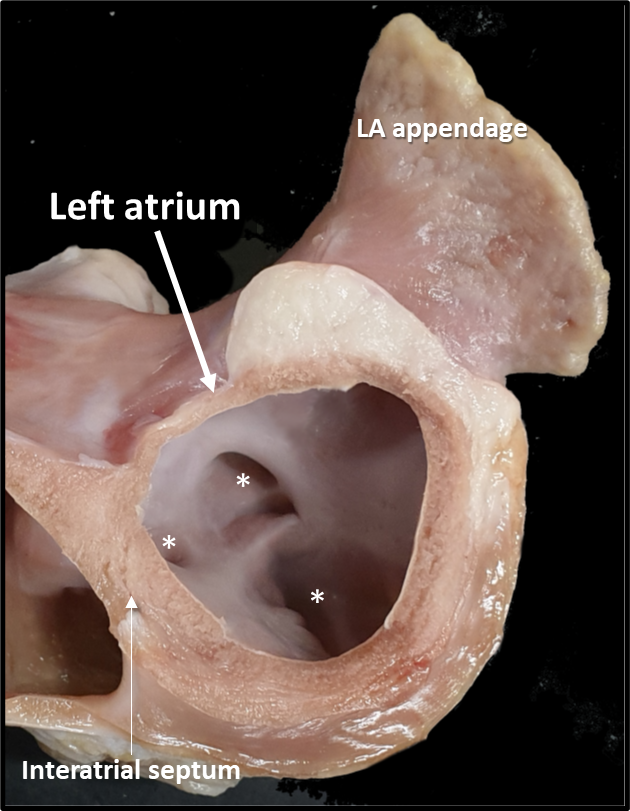


B. 4 months


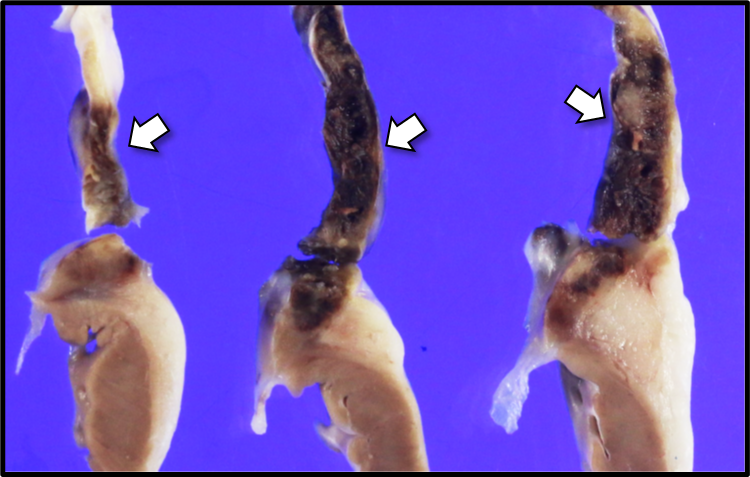


Gross findings of the left atrial myocardium from an irradiated dog heart with a circular box radiotherapy lesion (33 Gy). (A) The astericks (*) represent the opening of the pulmonary veins entering the left atrium. There is no visually damaged lesion in the myocardial area. (B) The white arrow indicates the irradiated target area in the left posterior wall.

**Supplementary figure S3**

**
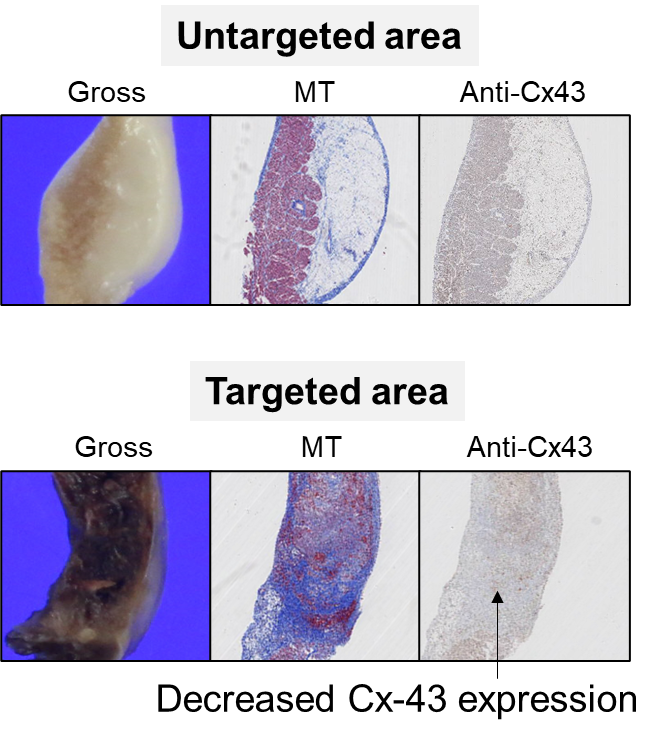
**

**MT,** Masson’s trichrome; **Cx-43,** Connexin-43


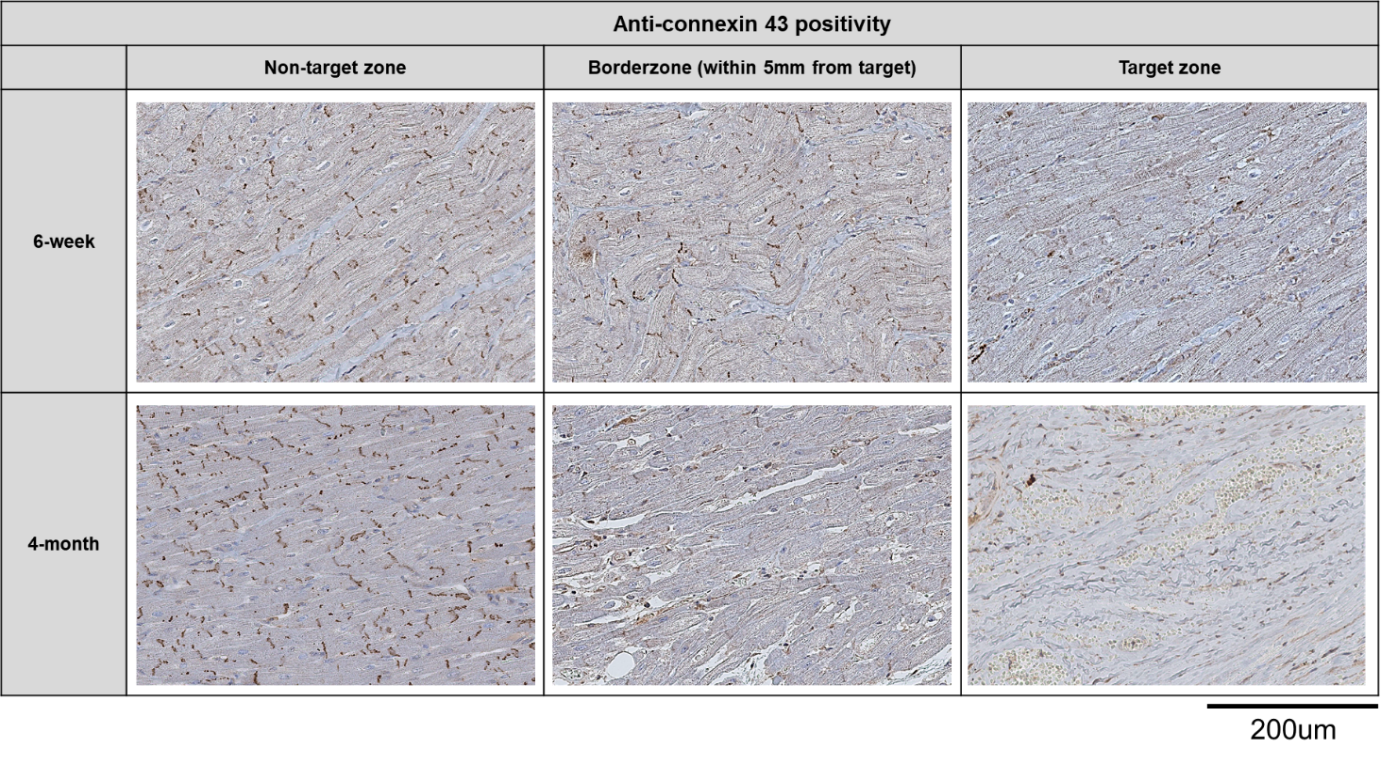


**Supplementary figure S4.**


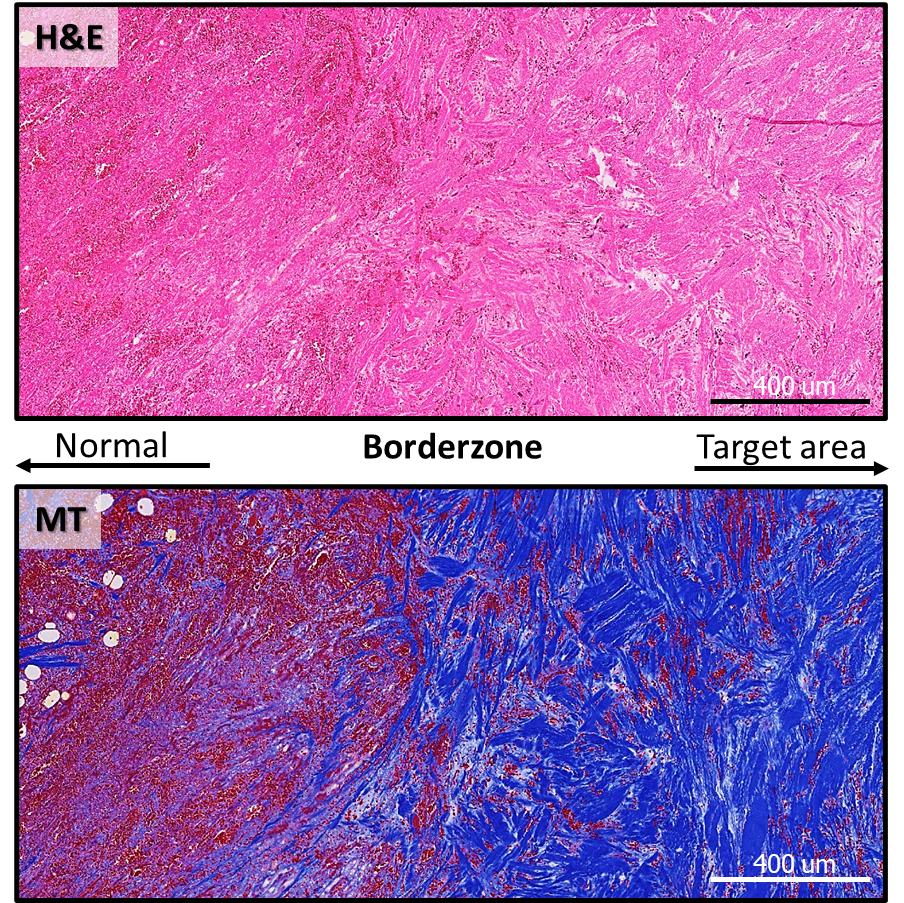


**Supplementary figure S5.**


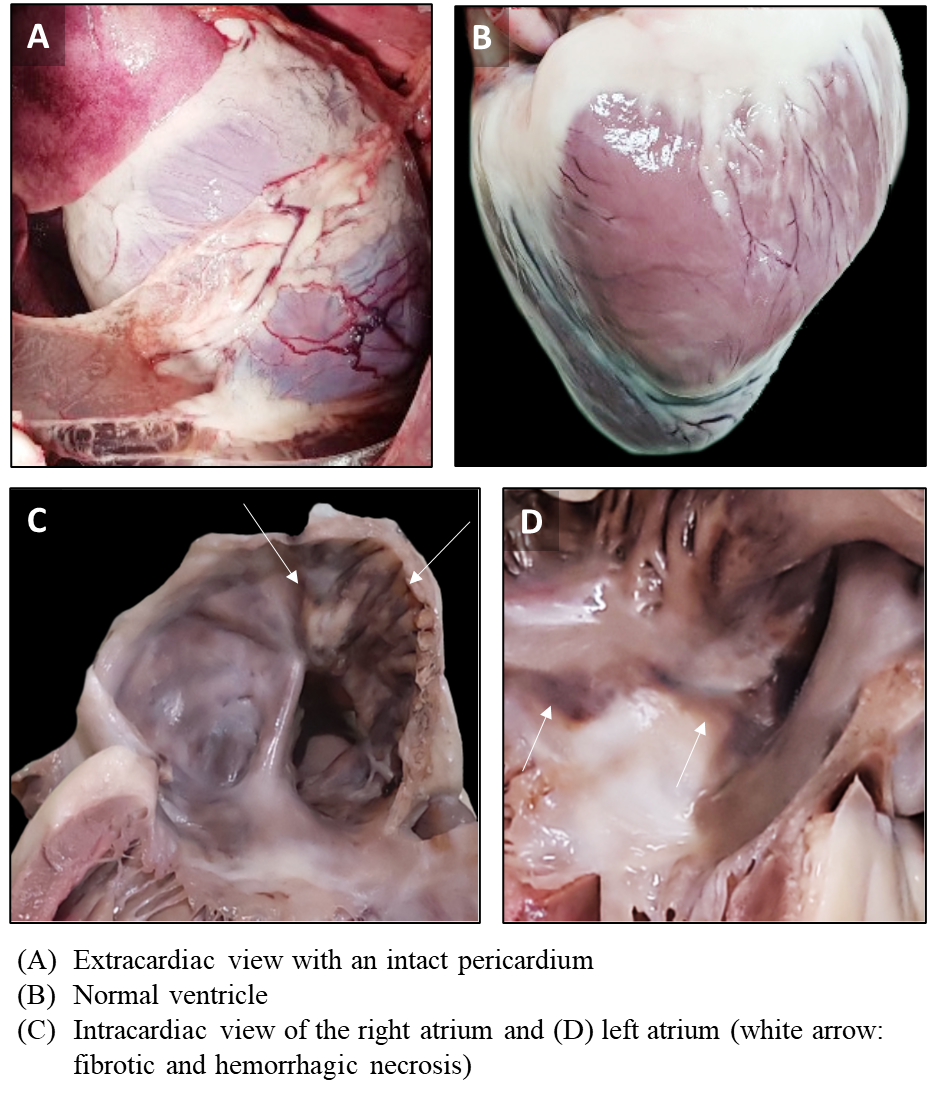


1. **Extracardiac view with an intact pericardium**
2. **Normal ventricle**
3. **Intracardiac view of the right atrium**
4. **left atrium** (white arrow: fibrotic and hemorrhagic necrosis)

**Supplementary figure S6.**

<Transient complete AV block during general anesthesia>


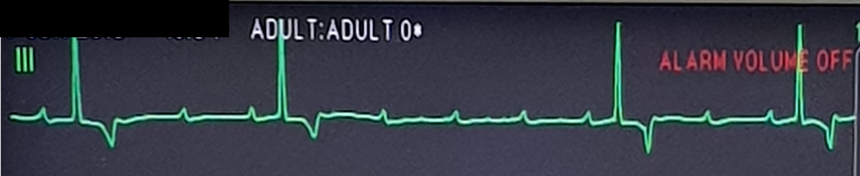
<Spontaneously recovered AV conduction>


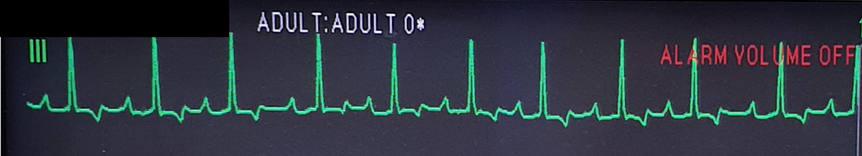

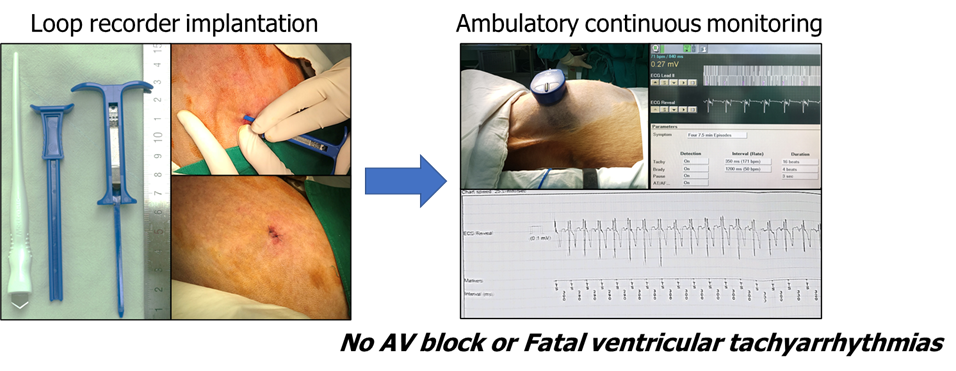


**Supplementary figure S7**


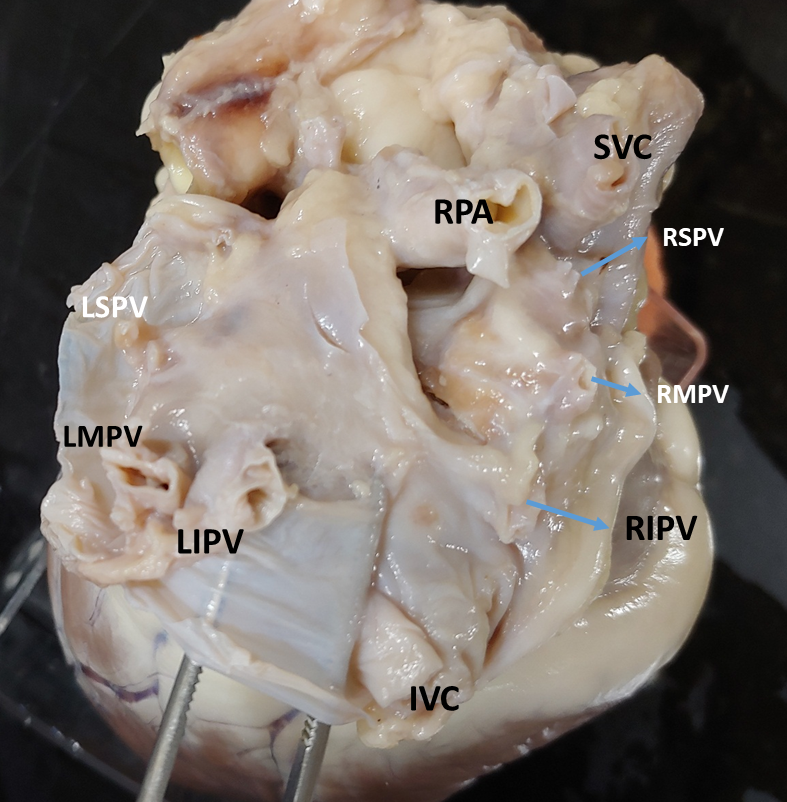


Posterior view of the atrial wall covered by pericardium. Forceps are introduced into the oblique sinus of the pericardium. The oblique sinus is shallow and covers only the inferior left part of the wall. Most of the posterior left atrial wall is covered by a broad fat pad. In this subject, we could find six ostia of pulmonary veins entering the left atrium.
